# Curcumin and Mesenchymal Stem Cells Ameliorate Ankle, Testis, and Ovary Deleterious Histological Changes in Arthritic Rats *via* Suppression of Oxidative Stress and Inflammation

**DOI:** 10.1155/2021/3516834

**Published:** 2021-11-09

**Authors:** Rania H. Ahmed, Sanaa R. Galaly, Nadia Moustafa, Rasha Rashad Ahmed, Tarek M. Ali, Basem H. Elesawy, Osama M. Ahmed, Manal Abdul-Hamid

**Affiliations:** ^1^Cell Biology, Histology and Genetics Division, Department of Zoology, Faculty of Science, Beni-Suef University, P.O. Box 62521, Beni-Suef, Egypt; ^2^Department of Physiology, College of Medicine, Taif University, P.O. Box 11099, Taif 21944, Saudi Arabia; ^3^Department of Pathology, College of Medicine, Taif University, P.O. Box 11099, Taif 21944, Saudi Arabia; ^4^Physiology Division, Zoology Department, Faculty of Science, Beni-Suef University, P.O. Box 62521, Beni-Suef, Egypt

## Abstract

Rheumatoid arthritis (RA) is a chronic inflammatory condition, an autoimmune disease that affects the joints, and a multifactorial disease that results from interactions between environmental, genetic, and personal and lifestyle factors. This study was designed to assess the effects of curcumin, bone marrow-derived mesenchymal stem cells (BM-MSCs), and their coadministration on complete Freund's adjuvant- (CFA-) induced arthritis in male and female albino rats. Parameters including swelling of the joint, blood indices of pro-/antioxidant status, cytokines and histopathological examination of joints, and testis and ovary were investigated. RA was induced by a single dose of subcutaneous injection of 0.1 mL CFA into a footpad of the right hind leg of rats. Arthritic rats were treated with curcumin (100 mg/kg b.wt./day) by oral gavage for 21 days and/or treated with three weekly intravenous injections of BM-MSCs (1 × 10^6^ cells/rat/week) in phosphate-buffered saline (PBS). The treatment with curcumin and BM-MSCs singly or together significantly (*P* < 0.05) improved the bioindicators of oxidative stress and nonenzymatic and enzymatic antioxidants in sera of female rats more than in those of males. Curcumin and BM-MSCs significantly (*P* < 0.05) improved the elevated TNF-*α* level and the lowered IL-10 level in the arthritic rats. Furthermore, joint, testis, and ovary histological changes were remarkably amended as a result of treatment with curcumin and BM-MSCs. Thus, it can be concluded that both curcumin and BM-MSCs could have antiarthritic efficacies as well as protective effects to the testes and ovaries which may be mediated *via* their anti-inflammatory and immunomodulatory potentials as well as oxidative stress modulatory effects.

## 1. Introduction

Rheumatoid arthritis (RA) is the most severe destructive inflammatory arthritis. It is a chronic autoimmune condition through which nonsuppurative proliferative synovitis contributes to destruction of the articular cartilage and bone resulting in multiple joint inflammation. RA is more common among women than among men [1, 2]. The severity of the disease ranges from person to person, with joint damage varying from mild pain and irritation to severe inflammation. RA also affects joint pairs (two hands, two feet) and can affect small joints in wrists and hands. Many joints such as knees, elbows, shoulders, feet, and ankles can be also affected over time and deformity occurs. In addition, other organs such as the skin, eyes, and lungs can be affected, and neuropathy, anemia, fatigue, and heart disease may occur [3]. Although the etiology of RA is unclear, disease susceptibility is associated with inheritance of certain allelic types of major histocompatibility complex (MHC) class II genes [4].

The mechanism of the joint degeneration effects in rheumatoid arthritis involves direct cell damage by cytotoxic CD^8+^ T-cells or other lytic cells. On other hand, the damaging effects of cytokines are triggered by CD^4+^ T-cells which know their antigenic targets, or by non-T-cells which release inflammatory mediators like tumor necrosis factor-*α* (TNF-*α*) and interleukin- (IL-) 1*β* [5]. In addition, Ahmed [6] suggested that the cytokine imbalance of CD^8+^ and CD^4+^ Th1/Th2 with a predominance of Th1 cytokines has pathogenic importance. TNF-*α*, a proinflammatory Th1 cytokine, serves a key role in the pathophysiological processes of RA [7, 8]. It is mainly released from activated inflammatory cells including macrophages, T-lymphocytes, and natural killer cells [9]. It contributes to the stimulation of other inflammatory cytokines, including interleukin- (IL-) 1, 6, 8, and 17 [7, 10]. TNF-*α* and other proinflammatory cytokines potentially amplify differentiation and activation of osteoclasts which in turn induce synovial hyperplasia, angiogenesis, cartilage erosion, and bone damage [11–13]. On the other hand, Th2 cytokines including IL-4 and IL-10 have anti-inflammatory effects, and their increases results in improving inflammation and arthritis [14, 15].

Reactive oxygen species (ROS) often participate in the pathogenesis of different diseases, including RA. ROS also play a central role both upstream and downstream of the TNF-*α* and nuclear factor-kappa B (NF-*κ*B) pathways, which are at the center of the inflammatory response. RA-related inflammation is associated with altered signaling pathways, resulting in elevated levels of inflammatory cytokine markers, lipid peroxides, and free radicals. The natural protection mechanism involves antioxidant enzymes like catalase (CAT), superoxide dismutase (SOD), and glutathione peroxidase (GPx), as well as nonenzymatic antioxidant and reduced glutathione (GSH). The defect in such protective mechanism contributes to toxic oxidative free-radical accumulation and consequent degenerative changes [14, 16]. Due to the adverse effects and toxicity arising from the use of antiarthritic drugs, more focus is placed in discovering safer, more efficient, natural product-based, alternative medicines with antioxidant activities [17–19].

Curcumin or diferuloylmethane is a polyphenolic yellow pigment derived from turmeric (*Curcuma longa*) and has been reported to exhibit numerous activities including antioxidant and anti-inflammatory properties [20, 21]. Curcumin is insoluble in water and ether but soluble in ethanol, dimethylsulfoxide, 1% carboxymethyl cellulose, and acetone [20, 22]. The fact that curcumin in solution exists primarily in its enolic form has an important role in the radical-scavenging ability of curcumin [20]. Many chronic disorders, including inflammatory arthritis, intestinal disease, chronic anterior uveitis, pancreatitis, and malignancies may benefit from curcumin [20]. Curcumin has also been shown to decrease many proinflammatory cytokines and their release mediators such as nitric oxide synthase (NOS), interleukin-8 (IL-8), interleukin-1 (IL-1), and TNF-*α* [21, 22].

The mesenchymal stem cell (MSC) population mainly resides in the bone marrow but may be present in other tissues (e.g., fat) and are capable of multilineage differentiation and self-renewal [23]. Under appropriate stimulation, MSCs can differentiate into 3 mesenchymal lineages: chondrocytes, adipocytes, and osteoblasts [23]. MSCs can also be induced experimentally to differentiate into neural and myogenic cells [24]. Multiple publications have confirmed that adherent cells (MSCs) isolated from various tissues meet the minimal criteria corresponding to the basic MSC phenotype, such as the expressions of CD73, CD90, and CD105 [25]. However, MSCs derived from different tissues can also express mesenchymal, hematopoietic, and endothelial tissue developmental markers [26], and they also produce molecules which directly involve immune response regulation, like programmed death ligand 1 (PDL-1) and PDL-2 inhibitory molecules, the costimulatory molecule CD28, and different cytokine arrays [27]. Therefore, MSCs can control immune response to these molecules. *In vivo*, MSC immunoregulatory function has also been observed; treatment with MSCs in humans enhanced the outcome of allogeneic transplantation through reducing graft-versus-host disease (GVHD) and facilitating hematopoietic engraftment [28]. MSCs have been widely used in animal models to prevent the autoimmunity recurrence in lupus-pronounced mice [29], to promote improvement of experimental autoimmune encephalomyelitis [30], and to enhance amelioration of CFA-induced arthritis in rats [14, 15]. Due to the success of MSC therapy in the treatment of some autoimmune disorders in animal models [30] and humans [28], the current research is aimed at examining the potential of bone marrow-derived mesenchymal stem cells (BM-MSCs) either singly or in combination with curcumin in the therapy of RA in male and female Wistar rats.

## 2. Materials and Methods

### 2.1. Chemicals

Complete Freund's Adjuvant (CFA) (10 mL; each 1 mL of CFA contains 1 mg of *Mycobacterium tuberculosis*, heat-killed and dried, 0.85 mL paraffin oil, and 0.15 mL mannide monooleate) was obtained from Sigma-Aldrich Chemical Co. (St. Louis, MO, USA). Curcumin was obtained from Hedel–De Han AG, Germany. DMEM (Dulbecco's modified Eagle's medium), trypsin/EDTA, penicillin-streptomycin solution, and fetal bovine serum (FBS) were obtained from Lonza, Belgium. Sodium hydrogen carbonate was obtained from LOBA Chemie, India. Culture flasks and culture consumables were obtained from Greiner Bio-One (Germany). Other reagents and all chemicals used were of analytical quality and high purity.

### 2.2. Preparation of Complete Culture Medium

To prepare the complete culture medium, 10 mL FBS and 1 mL penicillin-streptomycin solution were added to 89 mL DMEM for each preparation according to Sun et al. [31] and Ahmed et al. [32].

### 2.3. BM-MSC Isolation and Culture

The isolation and culture method of BM-MSCs used in this study is based on the procedure of Ahmed et al. [32] and Chaudhary and Rath [33]. Also, the viability of cultured cells are tested by staining with trypan blue (0.4%), and it was found to be 96-98%.

### 2.4. Experimental Animals

The used experimental animals in this study were randomly bred 48 adult males and 48 females of laboratory albino rats of Wistar strain weighing 110-150 g. The animals were delivered from the Experimental Animals Helwan Station, Egyptian Organization for Biological Products and Vaccines (VACSERA), Helwan, Cairo, Egypt. The animals were held in plastic cages that have wired covers and kept in normal laboratory conditions during the course of the experiment. The animals were not treated with antibiotics, insecticides, or vitamins and were fed a standard commercial diet (ATMID Company, Giza, Egypt) and tap water *ad libitum*. All experimental procedures were performed in accordance with recommendations, instructions, and guidelines stated by the Ethics Committee for Care and Use of Animals, Faculty of Science, Beni-Suef University, Egypt (Ethical Approval Number: BSU/FS/2018/7).

### 2.5. Experimental Design

**Figure 1:**
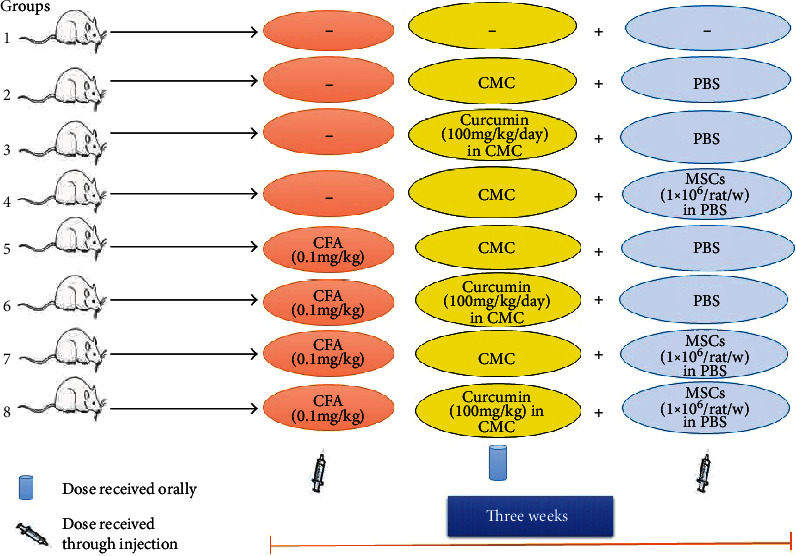
Experimental design and animal grouping.

Experimental animals (Figure 1) were organized into 16 groups (6 animals for each), eight groups including male rats and the other eight groups including female rats as follows:

1. Group 1: normal group that did not receive any treatment or vehicle.
2. Group 2 (control group): rats within this group received the equivalent volumes of 1% CMC (5 mL/kg b.wt./day) as vehicle 1 by oral gavage daily and PBS (as vehicle 2) in the lateral tail vein weekly for three weeks. The equivalent volume of phosphate-buffered solution was given.
3. Group 3: curcumin control group. Rats were daily supplemented with curcumin by oral gavage. Curcumin was dissolved in 1% CMC (carboxymethyl cellulose) at 2% concentration and was administrated orally (100 mg/kg b.wt./day) [34]. This group was also weekly given the equivalent volume of PBS.
4. Group 4 (mesenchymal stem cells (MSCs) control group): in this group, the rats weekly received injection of MSCs (1 × 10^6^/rat) in PBS. This group was daily given the equivalent volume of 1% CMC by oral gavage for 21 days.
5. Group 5 (arthritic control group): rats were subcutaneously injected with CFA (0.1 mL (0.1 mg)/kg b.wt. single dose) into a foot pad of the right hind leg [35] to induce RA. This group was also given the equivalent volumes of 1% CMC by daily oral administration and PBS by weekly intravenous injection.
6. Group 6 (arthritic group treated with curcumin): rats were injected with CFA like in group 5 and orally treated with curcumin like in group 3. This group was also weekly given the equivalent volume of PBS by intravenous injection.
7. Group 7 (arthritic group treated with BM-MSCs): in this group, rats were injected with CFA like group 5 in parallel to injection of BM-MSCs in the tail vein like group 4. This group was daily given the equivalent volume of 1% CMC by oral gavage for 21 days.
8. Group 8 (arthritic group treated with both MSCs and curcumin): rats were injected with CFA (like group 5) in parallel to oral administration of curcumin like groups 3 and 6 and injection of BM-MSCs in the tail vein like groups 4 and 7.

### 2.6. Tissues Sampling

The ankle circumference of the right hind leg of each rat was measured at the end of the experiment, and rats were sacrificed under mild anesthesia in each group. The ankle circumference was measured by wrapping a cotton thread around the ankle, and the length of the wrapped thread was measured by ruler. By centrifugation of blood at 3000 rpm for 15 minutes, sera were separated and the clear and nonhemolyzed supernatant sera were rapidly removed and kept at -20°C while being used in biochemical analysis. For histopathological analysis, paw and hind ankle, testes, and ovaries were removed and then fixed in neutral-buffered formalin (10%).

### 2.7. Paw Edema Level

The circumference of the right hind paw above the tarsal pad was determined by using a piece of cotton thread and wrapping it around the paw just above the tarsal pad as an indicator of the swelling rate and paw edema in different groups. The circumference was measured using a meter ruler [18, 36]. The measurements were taken on the 21th day of adjuvant induction.

### 2.8. Oxidative Stress Markers

In serum, the thiobarbituric acid-reactive substances (TBARS) were measured according to Preuss et al. [37] to determine lipid peroxidation (LPO). Glutathione reduced form (GSH) level was measured colorimetrically using the Ellman reagent as protein-free sulfhydryl content [38]. In addition, glutathione-S-transferase (GST) activity was calculated according to Habig et al. [39], and glutathione peroxidase (GPx) activity was determined by using the method of Kar and Mishra [40] in serum. Finally, superoxide dismutase (SOD) activity was detected according to the colorimetric method of Nishikimi et al. [41].

### 2.9. Detection of Serum TNF-*α* and IL-10 Levels

TNF-*α* levels in the serum of normal and experimental groups were measured using ELISA kits which were purchased from R&A Systems, USA, according to the manufacturer's instructions [42]. The level of IL-10 was determined using specific ELISA kits purchased from R&A Systems, USA, in the serum of control and experimental groups. According to the manufacturer's instructions, the concentrations of IL-10 were measured by using a spectrophotometer at 450 nm.

### 2.10. Histological Preparations

#### 2.10.1. Paraffin Section Preparation

Hind ankle region and paw tissue samples from male and female rats, testes, and ovaries were fixed for 24 h in 10% neutral-buffered formalin (pH 6.8). Tissue samples were embedded in paraffin wax after dehydration, sectioned at 4 to 5 *μ*m, and finally stained with hematoxylin and eosin for histopathological analysis [43].

### 2.11. Statistical Analysis

Two-way analysis of variance (ANOVA) [44] accompanied by one-way ANOVA using the SPSS/PC program (version 20.0; SPSS, Chicago, IL) and post hoc LSD test was used to statistically analyze biochemical variable measurements (*P* < 0.05 was considered to be significant). Two-way ANOVA was applied to assess the effects of gender, treatment, and gender/treatment interaction.

## 3. Results

### 3.1. Effect on Paw Edema

The right hind leg circumference, at the paw region in CFA-injected animals at day 21 of CFA injection and as a result of curcumin and MSC treatments, is shown in Tables 1 and 2.

The CFA-induced arthritic male and female rats exhibited a significant increase in the hind paw edema at day 21 as compared with the normal group. The arthritic effect in female rats is more severe than in male rats. The groups of the arthritic male and female rats treated with curcumin, MSCs, and their combination showed a significant amelioration of the elevated values of paw edema as compared to the arthritic animals and the values returned to nearly normal (Table 2).

Regarding one-way ANOVA, the general effect was very highly significant between groups (*P* < 0.001) (Table 1) throughout the experiment.

Concerning two-way ANOVA, it was noticed that the effect of treatment, gender, and treatment-gender interaction was significant at *P* < 0.001 (Table 1).

### 3.2. Effect on TNF-*α* and IL-10 Serum Inflammatory Cytokine Levels

The data showing the pattern of changes in serum TNF-*α* and IL-10 levels are represented in Tables 3, 4, 5, and 6.

A significant elevation in serum TNF-*α* level was noticed in CFA-induced arthritic rats when it was compared with normal rats; the arthritic effect is more severe in female than in male rats. CFA-injected rats that were treated with curcumin and/or MSCs exhibited a marked decrease in the elevated serum TNF-*α* level in comparison with arthritic control rats and when compared with either the curcumin- or MSC-treated arthritic groups (Table 4). Regarding one-way ANOVA, the general effect between groups on serum TNF-*α* level was highly significant (*P* < 0.001) (Table 3) throughout the experiment. Concerning two-way ANOVA, it was revealed that the effects of treatment, gender, and treatment-gender interaction were very highly significant at *P* < 0.001 (Table 3).

A significant decrease in serum IL-10 level was shown in CFA-induced arthritic rats when compared with normal rats after 21 days; the decrease is more pronounced in female than in male rats. The treatment of CFA-injected rats with MSCs and/or curcumin produced a significant increase in IL-10 level after 21 days in comparison to arthritic control rats; the combinatory effects were the most potent (Table 6). Regarding one-way ANOVA, the general effect between groups on serum IL-10 level was very highly significant (*P* < 0.001) (Table 5) throughout the experiment. Concerning two-way ANOVA of normal-arthritis effect, it was revealed that the effects of treatment and treatment-gender interaction were very highly significant (*P* < 0.001), while the effect of gender is only significant (*P* < 0.05) (Table 5).

### 3.3. Oxidative Stress Markers

The data showing the effects on LPO, GSH content, and antioxidant enzymes in serum are represented in Tables 7, 8, 9, 10, 11, and 12.

Table 12 shows changes in LPO and antioxidant parameters for all groups. MDA level, as an indicator of LPO, exhibited a significant increase (*P* < 0.05) in male and female rats in comparison with the normal group. CFA injection resulted in MDA level increase in female rats more than in male rats. On the other hand, the treatment with curcumin, MSCs, and a mixture of both induced a potential reduction of elevated MDA in both male and female rats.

Concerning the two-way ANOVA. The MDA level of arthritic rats treated with curcumin, MSCs, and combination effects, it was noticed that the effects of treatment and gender were very highly significant (*P* < 0.001), while the effects of gender-treatment interaction were nonsignificant (*P* > 0.05) (Table 7).

The level of the nonenzymatic antioxidant, GSH, and the activities of antioxidant enzymes including GST, GPx, and SOD showed a significant depletion in CFA-induced arthritic rats. On the other hand, the treatment with curcumin, MSCs, and their combination induced a significant improvement in GSH, GST, GPx, and SOD activities in both male and female rats; the effects of curcumin, MSCs, and their combination were more or less similar.

Concerning the one-way ANOVA, CFA caused significant effects in GSH levels (Table 8) along with GST (Table 9), GPx (Table 10), and SOD (Table 11) activities (*P* < 0.001) in both male and female rats compared to the normal group.

Concerning the two-way ANOVA, in the case of GSH content, it was noticed that the effects of treatment were very highly significant (*P* < 0.001), and the effects of gender were significant (*P* < 0.05), while the effects of gender-treatment interaction were nonsignificant (*P* > 0.05) (Table 8). In the case of GST and GPx activities, it was noticed that the effects of gender and gender-treatment interaction were significant (*P* < 0.05), and the effects of treatment were very highly significant (*P* < 0.001) (Table 9). Finally, in the case of SOD, it was revealed that the effects of gender were significant (*P* < 0.05), and the effects of treatment were significant (*P* < 0.001), while the effects of treatment-gender interaction were nonsignificant (*P* > 0.05) (Table 10).

### 3.4. Histopathological Results

**Figure 2:**
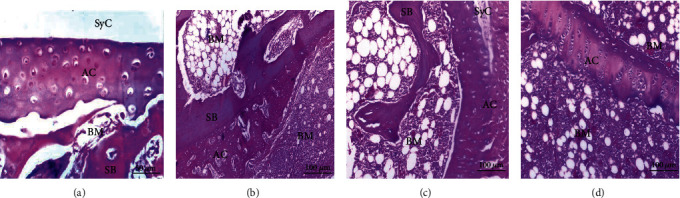
Photomicrographs of sections in the ankles of male rats from control groups: (a) water, (b) CMC, (c) curcumin, (d) stem cells showing normal articulating cartilage (AC), synovial cavity (SyC), sponge bone (SB), and bone marrow (BM) (H&E).

**Figure 3:**
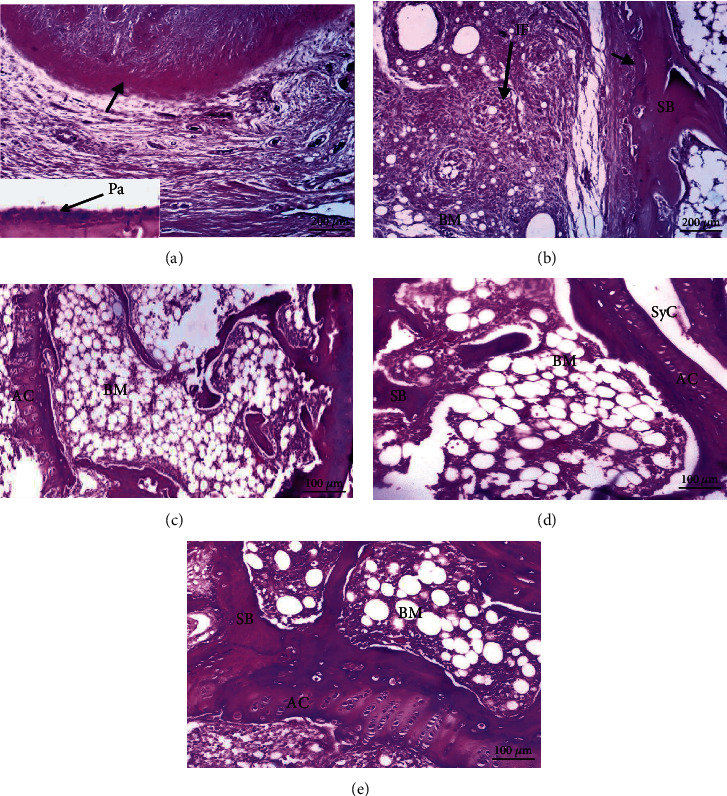
Photomicrographs of sections in the ankles of male rats. (a) From the RA group showing necrosis of articular cartilage (↗). The insert shows pannus formation (↙). (b) From the RA group showing necrosis of articular cartilage (↓) and inflammatory cell infiltration (IF). (c–e) From arthritic-treated groups with curcumin, MSCs, and a combination of curcumin and MSCs, respectively, showing a nearly normal structure of articulating cartilage (AC), synovial cavity (SyC), sponge bone (SB), and bone marrow (BM).

**Figure 4:**
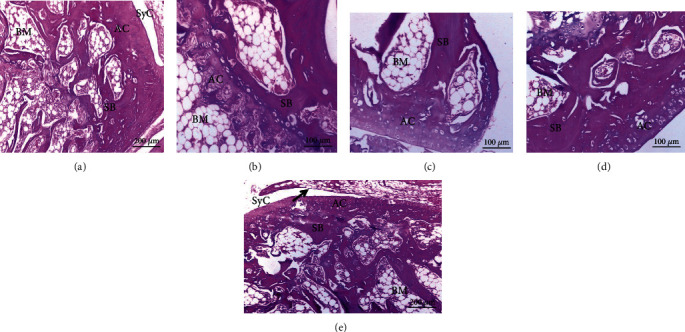
Photomicrographs of sections in the ankles of female rats from control groups: (a, b) water; (c) CMC; (d) curcumin; (e) stem cells showing normal articulating cartilage (AC), synovial cavity (SyC), synovial membrane (↗), sponge bone (SB), and bone marrow (BM).

**Figure 5:**
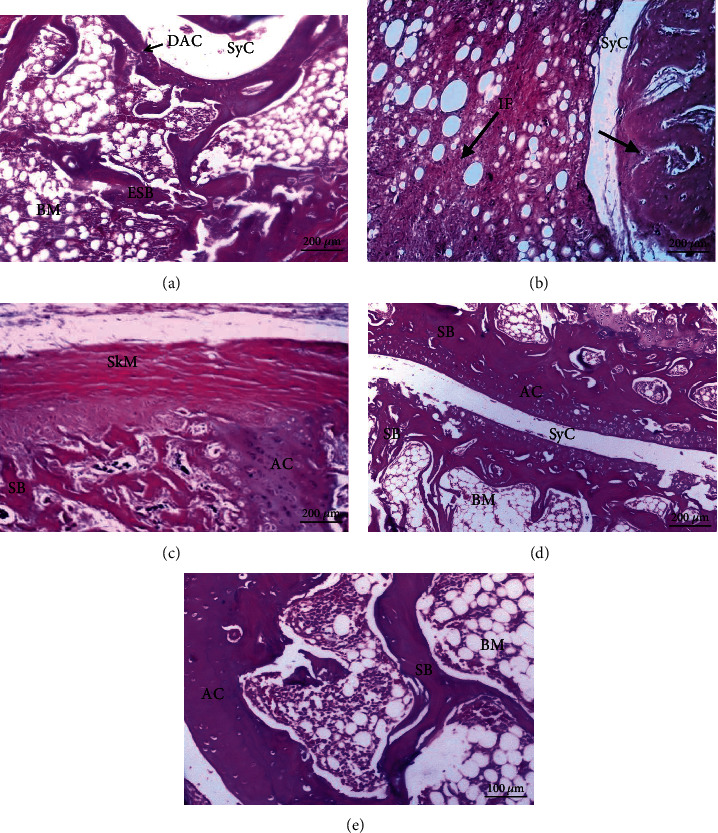
Photomicrographs of sections in an ankle of a female rat (a) from the RA group showing damaged articulating cartilage (DAC) and eroded sponge bone (ESB); (b) from the RA group showing necrosis of articular cartilage (↘) and massive inflammatory cell infiltration (IF); (c–e) from arthritic-treated groups with curcumin, MSCs, and a mixture of curcumin and MSCs, respectively, showing a nearly normal structure of articulating cartilage (AC), synovial cavity (SyC), skeletal muscle (SkM), sponge bone (SB), and bone marrow (BM).

The histological alterations of the articular ankle joint in various groups of male and female rats are depicted in Figures 2–5. In normal rats, the bone surfaces in the synovium are covered by an articular cartilage that lacks a perichondrium. The heads of the two articulated bones are enclosed and joined by an articular capsule consisting of two parts, the outer and inner parts. The outer part is a sheath of fibrous tissue (fibrous capsule) that extends well beyond each bone's articular cartilage. The inner part is called a synovial membrane and lines the fibrous capsule and is reflected in the bone that covers right up to the articular cartilage. Therefore, the joint cavity between two articulated bones is lined everywhere with either articular cartilage or synovial membrane. The synovial membrane is a thin sheath of fibrous connective tissue, with a dense network of blood and lymph capillaries. Ankle joint sections of male rats (Figures 2(a)–2(d)) and female rats (Figures 4(a)–4(e)) from normal, CMC, and combined curcumin and MSC groups, respectively, showed the normal histological structure of an ankle with normal articulating cartilage, synovial cavity, sponge bone, and bone marrow.

CFA-administered arthritis male rats showed necrosis of cartilage with inflammatory cell infiltration in ankle joint sections, degeneration of cartilage, and pannus formation (Figures 3(a) and 3(b)).

CFA-administered arthritis female rat ankle joint sections showed severe necrosis of cartilage with massive inflammatory cell infiltration, severe degeneration of cartilage, and eroded spongy bone (Figures 5(a) and 5(b)). This indicated that the arthritic effect was more severe in female rats than in male rats.

CFA-administered male rats (Figures 4(c)–4(e)) and female rats (Figures 5(c)–5(e)) treated with curcumin, MSCs, and a mixture of curcumin and mesenchymal stem cells showed a nearly normal section structure of articulating cartilage, synovial cavity, sponge bone, and bone marrow nearly similar to the normal control groups.

**Figure 6:**
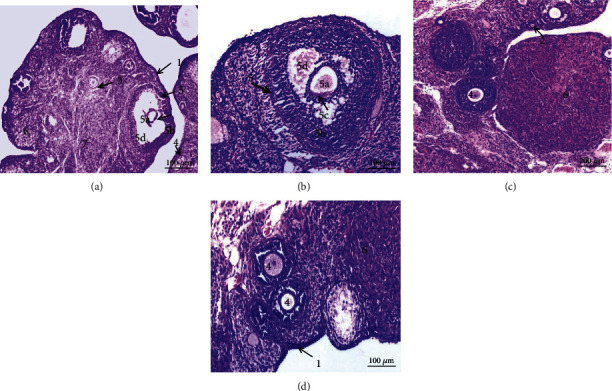
Photomicrographs of control rat ovarian sections: (a) water; (b) CMC; (c) curcumin; (d) stem cells showing normal histological structure. (1) Germinal epithelium; (2) primordial follicles; (3) primary follicles; (4) secondary follicles; (5) mature (antral or Graafian) follicle including (5a) oocyte (immature ovum), (5b) granulosa cells (stratified cuboidal epithelium), (5c) zona pellucida, and (5d) antrum; (6) corpus luteum; (7) medulla (H&E).

The ovary of control normal rats (Figures 6(a)–6(d)) from normal, CMC, curcumin, and MSCs, respectively, showed a normal morphology. The ovary consists of two distinct regions: an outer cortex that contains numerous follicles at various stages of maturation and an inner central medulla, which did not appear in these histological sections. The surface of the ovary is covered with germinal epithelium. It contains corpus luteum and different primordial follicles including primary follicles and secondary follicles. The secondary follicle contains an oocyte surrounded by two or more layers supporting granulosa cells and a follicular antrum filled with liquor follicle, and the follicle is surrounded by theca interna. Mature Graafian follicles are seen beneath the epithelium. Graafian follicles consist of an enlarged oocyte that floats freely within liquor folliculi surrounded by clear zona pellucida, corona radiata, well-defined zona granulosa, and compact theca folliculi.

**Figure 7:**
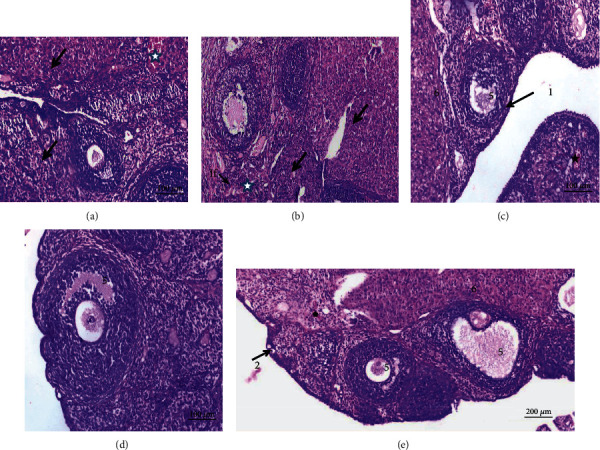
Photomicrographs of rat ovarian sections (a, b) from the RA group showing numerous luteal structures (arrow) in ovarium medulla and hyperemia in the stroma (star); (c–e) from arthritic-treated groups with curcumin, stem cells, and a mixture of curcumin and stem cells, respectively, showing nearly normal histological structure except for a few hyperemic areas in the stroma (star) and mononuclear cell infiltration (IF). (1) Germinal epithelium, (2) primordial follicles, (5) mature (antral or Graafian) follicle, and (6) corpus luteum (H&E).

The histopathological examination of arthritic ovarian sections revealed multiple luteal structures in ovarium medulla, stromal hyperemia, and infiltration of mononuclear cells (Figures 7(a) and 7(b)).

Sections of arthritic rats treated with curcumin (Figure 7(c)), MSCs (Figure 7(d)), and a combination of curcumin and mesenchymal stem cells (Figure 7(e)) revealed nearly normal structure.

**Figure 8:**
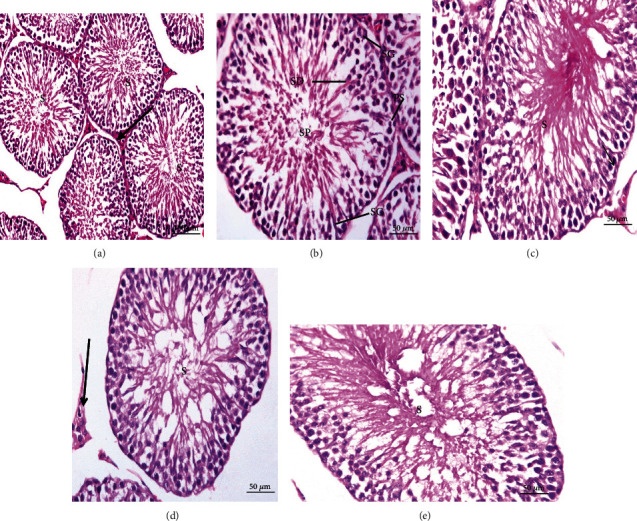
Photomicrographs of sections in testis of control rat groups: (a) water; (b) magnified cross section of (a); (c) CMC; (d) curcumin; (e) stem cells showing normal histological structure seminiferous tubules (S), interstitial space containing Leydig cells (arrow), spermatogonia (SG) resting upon the basal lamina of the seminiferous tubules that are oval in shape, primary spermatocytes (PS), recognized by their large nuclei, spermatids (SD) with rounded nuclei, Sertoli cells (SC) with nuclei located basally, and sperms in the lumen of the tubules (SP) (H&E).

Testes of normal rats (Figures 8(a) and 8(b)), CMC (Figure 8(c)), curcumin (Figure 8(d)), and stem cells (Figure 8(e)) revealed a normal seminiferous tubule morphology. Every tubule has epithelial cells including Sertoli cells and germ cells that demonstrated the complete spermatogenesis process (Figure 8(b)). Sertoli cells were usually located in the seminiferous tubule toward the basement membrane. Spermatogonia stood on seminiferous tubule basal lamina. Primary spermatocytes were immediately above them, identified by their large nuclei having coarse chromatin clumps and copious cytoplasm. Due to the rapid division processes, secondary spermatocytes in these sections were not seen. Therefore, there were small, rounded spermatids with rounded nuclei above the primary spermatocytes that proceeded in a long metamorphosis to become recognizable spermatozoa (Figure 8(b)).

**Figure 9:**
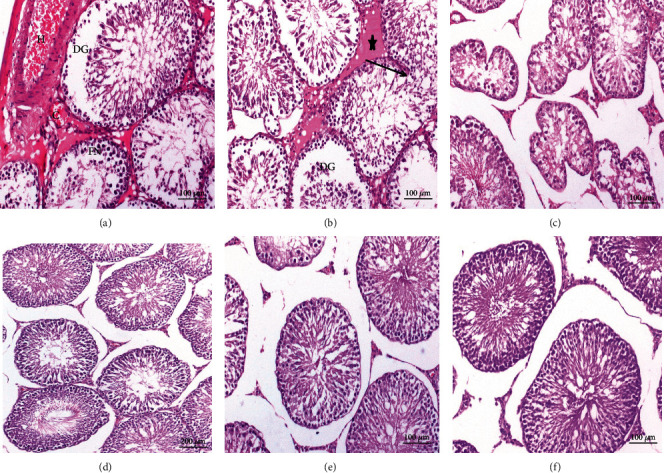
Photomicrographs of rat testis sections. (a) From the RA group showing capillary congestion (C), hemorrhage (H), and focal necrosis in germinal cells (FN). (b) From the RA group showing pyknotic nuclei (thin arrows) of primary spermatocytes (PS), interstitial edema (star), and degeneration of germ cells (DG). (c) From the RA group showing irregular variable-sized seminiferous tubules and atrophy in seminiferous tubules, with most tubules showing a marked decrease in the number of spermatogenic cells; consequently, the lumen of the tubules appeared with few or no sperms observed. (d–f) From arthritic-treated groups with curcumin, stem cells, and a mixture of stem cells and curcumin, respectively, showing a nearly normal histological structure of the seminiferous tubules (H&E).

Testicular tissue sections obtained from CFA-treated rats displayed several histopathological changes as showed in Figures 9(a)–9(c). Atrophy and focal necrosis in germinal cells, spermatogenic arrest, and congestion were noticeably observed (Figure 9(a)). Pyknotic nuclei, interstitial edema, and damaged seminiferous epithelium and germ cells are also seen (Figure 9(b)). The seminiferous tubules showed irregular variable size and congestion in intercellular space (Figure 9(c)). Testes treated with curcumin, MSCs, and mixture of MSCs plus curcumin (Figures 9(d)–9(f)), respectively, revealed apparent normal seminiferous tubules. Spermatogenic layers were well organized, the tubules had restored their regular shape, and sperms in most of the tubules were observed.

## 4. Discussion

Currently, stem cell therapy has been declared as one of the most important and promising treatments for the near future. This kind of therapy could improve or even reverse some degenerative diseases and have potential applications in replacement and regenerative medicines and RA. Also, using plant constituents in RA treatment has attracted many researchers due to the side effects of conventional drugs.

**Figure 10:**
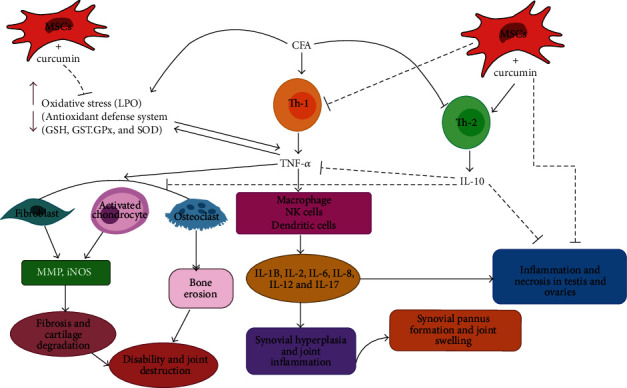
Schematic figure showing the mode of actions of CFA to induce arthritis *via* increasing LPO and TNF-*α* (Th1 cytokine) and decreasing IL-10 (Th2), thereby resulting in synovial hyperplasia and joint, testis, and ovary necrosis, and inflammation. The treatment with curcumin and/or MSCs can counteract these actions *via* enhancing the antioxidant defense system and anti-inflammatory mechanisms. →: activation; ┴: inhibition.

RA, one of the most common chronic inflammatory autoimmune diseases, is distinguished by systemic inflammation, permanent synovitis, edema, and production of autoantibodies [45]. Because of their multipotent differentiation abilities, cell therapy using MSCs is the most common new technique in tissue repair and regeneration [14, 15, 32, 46]. Additionally, MSCs have therapeutic potential to joint and bone diseases through the secretion of a number of immune modulating substances and cell-to-cell interactions leading to antiapoptotic, antifibrotic, immunosuppressive, and proangiogenic properties [47]. Curcumin, a polyphenolic yellow pigment derived from *Curcuma longa* Linn, is a member of the compound family curcuminoid. Curcumin, derived from diferuloylmethane, is an important antioxidant which has been used as herbal therapy and as a dietary factor in many Eastern countries. Curcumin has also been shown to inhibit many proinflammatory cytokines and mediators such as IL-1, IL-8, and nitric oxide synthase [48]. Consequently, curcumin's beneficial effects on inflammatory disorders are due to the suppression of immune functions of T-cells, specially Th1, which plays a key role in the pathogenesis of chronic inflammatory disorders such as arthritis (Figure 10) [14, 15, 18, 49].

In the present study, due to treatment with MSCs and curcumin, the increased right hind leg ankle joint circumference of male and female arthritic rats was significantly reduced. This decrease in the joint circumference of the ankle represents the swelling rate decrease that can be due to edema reduction, inflammatory process attenuation, and synovial tissue hyperplasia reduction as demonstrated by the histological results in the current study and stated by previous publications [22, 50].

Serum concentrations of TNF-*α* and IL-10 were determined in the current study to elucidate their potential anti-inflammatory roles in the mechanisms of action of curcumin and MSCs. The TNF-*α* serum proinflammatory cytokine was significantly elevated in arthritic rats, and the effect was more deteriorated in female than in male arthritic rats. The IL-10 serum level of anti-inflammatory cytokine was depleted in arthritic rats and also was more deteriorated in female than in male arthritic rats. Therefore, changes of these cytokines ensure that Th1 cytokines are dominant over Th2 cytokines (Figure 10). Many previous authors supported this evidence [22, 51].

In the present study, numerous histopathological changes in bone, ovarian, and testicular tissues were noticed in arthritic rats. The ankle joint of CFA-administered arthritic rats exhibited deleterious histological changes including necrosis, eroded articulating cartilage, and pannus formation [52]. These histopathological alterations may be attributed to the increase in the oxidative stress; antioxidant defense system suppression; elevation in the proinflammatory and inflammatory cytokines represented by increased IL-1*β*, IL-6, and COX-1 mRNA expression; and depletion of anti-inflammatory cytokines represented by decreased IL-4 mRNA expression. The improvement of ankle joint histological architecture as a result of treatment of arthritic rats with MSCs and curcumin may be due to their ability to scavenge lipid peroxides and free radicals, enhance the antioxidant defense system, and suppress inflammatory status. MSCs are able to inhibit osteoclast-mediated bone resorption, resulting in bone degradation through induction of T regs and reduction in the development of inflammatory cytokines that aid osteoclastogenesis. It has been demonstrated that osteoclastogenesis is inhibited by MSCs through production of osteoprotegerin or using interactions with osteoclast precursors via CD200/CD200 receptor interactions [53]. Garimella et al. [54] suggested that MSC injection into the collagen-induced arthritis (CIA) mice prevented bone loss *via* decreasing bone marrow osteoclast precursors but the mechanisms remain unclear.

Antioxidants are compounds which can delay, inhibit, or avoid oxidation of compounds, capture free radicals, and reduce oxidative stress. The body has an effective mechanism for preventing and neutralizing the free-radical-caused damage. This is accomplished by a group of endogenous antioxidant enzymes like SOD and CAT and the nonenzymatic antioxidant, GSH. Oxidative stress leads to cellular function deregulation that leads to different pathological conditions when the balance between ROS production and antioxidant defense is lost [55]. In rheumatoid arthritis, oxygen free radicals are implicated as tissue damage mediators. The involvement of free radicals is well studied in various inflammatory conditions, such as synovitis and rheumatoid arthritis. In the present study, the results revealed significant lipid peroxidation increase and decrease in antioxidant enzymes as well as GSH in male and female arthritic rats. Polyphenols have the ability of protecting cells from oxidative stress. However, polyphenol compounds may have antioxidant/prooxidant properties, depending on the source and concentration of free radicals [56]. The combination of curcumin and MSCs revealed a significant decrease of LPO when compared with the arthritic group. Significant normalization of the levels of antioxidant enzymes (GST, GPx, and SOD) and GSH promoted the potent antiarthritic curcumin activity with MSC combination. Arthritic rats treated with curcumin revealed a significant increase in GSH, GST, GPx, and SOD levels when compared to arthritic control. In this study, it was determined that administration of MSCs plus curcumin in arthritic rats significantly attenuated the changes in LPO, GPx, SOD, GST, and GSH. LPO was significantly reduced in arthritic rats treated with curcumin and MSCs as compared to the arthritic control, where all values approximately returned to the normal level.

It was shown in this study that the ovarian tissue of the arthritic female rats had multiple luteal structures in ovarium medulla, stromal hyperemia, and infiltration of mononuclear cells. According to Kim and Boone [57], at the penultimate stage of follicular development in the ovary, FasL is present in granulosa cells and may be the signal that causes apoptosis of granulosa cell during atresia.

In the present study, testicular changes include necrosis in germinal cells, pyknotic nuclei, interstitial edema, atrophy, vacuolation, and blood vessel congestion may be due to an increase of free radicals and elevation of inflammatory cytokines. *In vitro* studies on seminiferous tubule cultures revealed that IFN-*γ* and TNF-*α* caused germ cell apoptosis *via* the Fas-FasL system [58–60]. In the same regard, Rival et al. [61] also reported that IL-6 induced germ cell apoptosis.

The controls on the immune cells and inflammatory cytokines that are involved in RA are due to MSCs. The activation and proliferation of B-cells and T-lymphocytes are inhibited by MSCs *via* cytokine secretion (paracrine effect) and also a cell-cell direct contact effect [62, 63]; thus, they have a protective effect against ovarian and testicular tissue damage induced by RA.

Curcumin can ameliorate the destructive damage of testis and ovary tissues because of the ability to scavenge lipid peroxides and free radicals, enhance the antioxidant defense system, and suppress inflammatory status, which are elevated due to CFA that induce arthritis.

## 5. Conclusion

In conclusion, the present study shows that CFA induced oxidative stress and ankle, ovarian, and testicular damage not. The administration of curcumin and BM-MSCs singly or in combination provides potential protective activities against oxidative stress changes and articular inflammatory cell infiltration and ameliorates the histopathological effects of CFA male and female rats; the combinatory effects are more potent in both male and female arthritic rats. Consequently, we advise using the combination of mesenchymal stem cells and curcumin due to their antioxidant and anti-inflammatory properties and their ameliorating role in histopathological changes. However, clinical studies are required to assess the efficacy and safety of this combination before approval of its application for treatment in human beings.

## Figures and Tables

**Table 1:** Effect of gender, group and their interactions on paw circumference in rats (one-way and two-way ANOVA).

Source of variation		Sum of squares	D.F.	Mean squares	*F* ratio	*P* value
One-way ANOVA	General effect					
	In between groups	17.055	15	1.137	46.748	*P* < 0.001
	Within groups	1.946	80	0.024		
	Total	19	95			
Two-way ANOVA	Gender	0.196	1	0.196	8.067	*P* < 0.001
	Treatment	15.529	7	2.218	91.211	*P* < 0.001
	Gender-treatment	1.330	7	0.190	7.812	*P* < 0.001
	Error Total	1.946 110.925	80	0.024		

*P* < 0.001 is significant effect at *α* = 0.001.

**Table 2:** Effect of tested materials (MSCs and curcumin) on circumference of paw region (cm) of arthritic group.

Parameter	Right hind leg paw circumference at (cm)	
Group	Male rats	Female rats
Normal (water)	1.925 ± 0.017^a^	1.996 ± 0.016^a^
CMC/PBS	1.943 ± 0.018^a^	1.995 ± 0.020^a^
MSCs	1.978 ± 0.028^a^	2.028 ± 0.060^a^
Curcumin	1,986 ± 0.027^a^	1.998 ± 0.017^a^
Arthritic group (CFA)	3.856 ± 0.231^b^	9.500 ± 0.215^c^
Arthritic group treated with curcumin	2.023 ± 0.059^a^ 880.87	2.060 ± 0.097^a^ 1425.00
Arthritic group treated with MSCs	2.031 ± 0.084^a^ −72.87	2.018 ± 0.070^a^
Arthritic group treated with MSCs and curcumin	2.255 ± 0.192^a^	2.025 ± 0.022^a^

Data are expressed as mean ± SE. The number of animals in each group is six. Values with the same superscript letter are similar (nonsignificant, *P* > 0.05), whereas others are significant (significant, *P* < 0.05).

**Table 3:** One-way and two-way analysis for serum TNF-*α* (pg/mL) level in normal, arthritic, and arthritic-treated rats.

Source of variation		Sum of squares	D.F.	Mean squares	*F* ratio	*P* value
One-way ANOVA	General effect					
	In between groups	19177.342	16	1198.584	43.907	*P* < 0.001
	Within groups	2156.54	79	27.298		
	Total	21333.890	95			
Two-way ANOVA	Gender	395.444	1	395.444	14.415	*P* < 0.001
	Treatment	17417.571	7	2488.224	90.702	*P* < 0.001
	Gender-treatment	1326.246	7	189.464	6.906	*P* < 0.001
	Error Total	2149.629 470842.89	80	27.433		

*P* < 0.001indicates significant effect at *α* = 0.001.

**Table 4:** Effect of MSCs and curcumin on serum TNF-*α* (pg/mL) level in arthritic rats.

Parameter Group	TNF-*α* (pg/mL serum)	
Group	Male rats	Female rats
Normal	55.59 ± 1.48^a^	63.19 ± 5.06^a^
CMC/PBS	58.25 ± 0.88^a^	61.57 ± 0.91^a^
MSCs	64.84 ± 0.76^a^	56.47 ± 1.56
Curcumin	66.58 ± 1.20^a^	56.31 ± 3.14^a^
Arthritic group (CFA)	96.43 ± 1.31^b^	103.50 ± 3.37^c^
Arthritic group treated with curcumin	84.26 ± 1.85^d^	64.70 ± 1.73^a^
Arthritic group treated with MSCs	74.05 ± 1.29^d^-	72.16 ± 1.23^d^
Arthritic group treated with MSCs and curcumin	56.62 ± 1.57^a^	61.40 ± 2.44^a^

Data are expressed as mean ± SE. The number of animals in each group is six. Values with the same superscript letter are similar (nonsignificant, *P* > 0.05), whereas others are significant (significant, *P* < 0.05).

**Table 5:** One-way and two-way analysis for serum IL-10 (pg/mL) level in normal, arthritic and arthritic-treated rats.

Source of variation		Sum of squares	D.F.	Mean squares	*F* ratio	*P* value
One-way ANOVA	General effect					
	In between groups	15720.113	16	982.507	8.832	*P* < 0.001
	Within groups	8788.131	79	111.242		
	Total	24508.244	95			
Two-way ANOVA	Gender	477.265	1	477.265	4.341	*P* < 0.05
	Treatment	11886.638	7	1698.091	15.444	*P* < 0.001
	Gender-treatment	3348.098	7	478.300	4.350	*P* < 0.001
	Error Total	8796.243 897550.02	80	109.953		

*P* < 0.001indicates significant effect at *α* = 0.001, and *P* < 0.05 indicates significant effect at *α* = 0.05.

**Table 6:** Effect of MSCs and curcumin on serum IL-10 (pg/mL) level in arthritic rats.

Parameter Group	IL-10 (pg/mL)	
	Male rats	Female rats
Normal	104.96 ± 1.6^a^	103.44 ± 4.42^a^
CMC/PBS	104.33 ± 2.06^a^	102.16 ± 3.58^a^
MSCs	103.08 ± 3.39^a^	104.70 ± 7.18^a^
Curcumin	105.82 ± 4.53^a^	105.38 ± 3.34^a^
Arthritic group (CFA)	79.83 ± 2.17^b^	53.26 ± 1.00^c^
Arthritic group treated with curcumin	91.40 ± 6.32^a^	88.51 ± 4.62^a^
Arthritic group treated with MSCs	85.37 ± 6.38^a^	89.22 ± 4.94^a^
Arthritic group treated with MSCs and curcumin	96.25 ± 2.21^a^	97.53 ± 5.01^a^

Data are expressed as mean ± SE. The number of animals in each group is six. Values with the same superscript letter are similar (nonsignificant, *P* > 0.05), whereas others are significant (significant, *P* < 0.05).

**Table 7:** One-way and two-way analysis to test the effect of gender, treatment, and their interactions on MDA content in rats.

Source of variation		Sum of squares	D.F.	Mean squares	*F* ratio	*P* value
One-way ANOVA	General effect					
	In between groups	344.312	15	22.954	18.389	*P* < 0.001
	Within groups	99.858	80	1.248		
	Total	444.169	95			
Two-way ANOVA	Gender	48.920	1	48.920	38.403	*P* < 0.001
	Treatment	285.120	7	40.740	31.981	*P* < 0.001
	Gender-treatment	8.158	7	1.165	0.915	*P* > 0.05
	Error Total	101.910 2027.001	80	1.274		

*P* < 0.001indicates significant effect at *α* = 0.001, while *P* > 0.05 indicates insignificant.

**Table 8:** One-way and two-way analysis to test the effect of gender, treatment, and their interactions on GSH content in rats.

Source of variation		Sum of squares	D.F.	Mean squares	*F* ratio	*P* value
One-way ANOVA	General effect					
	In between groups	260.032	15	17.335	10.822	*P* < 0.001
	Within groups	128.145	80	1.602		
	Total	388.168	95			
Two-way ANOVA	Gender	6.531	1	6.531	4.079	*P* < 0.05
	Treatment	251.022	7	35.860	22.397	*P* < 0.001
	Gender-treatment	2.526	7	0.361	0.225	*P* > 0.05
	Error Total	128.089 4975.856	80	1.601		

*P* < 0.001indicates significant effect at *α* = 0.001, and *P* < 0.05 indicates significant effect at *α* = 0.05, while *P* > 0.05 is insignificant.

**Table 9:** One-way and two-way analysis to test the effect of gender, treatment, and their interactions on GST content in rats.

Source of variation		Sum of squares	D.F.	Mean squares	*F* ratio	*P* value
One-way ANOVA	General effect					
	In between groups	1096.462	15	73.097	14.362	*P* < 0.001
	Within groups	407.168	80	5.090		
	Total	1503.630	95			
Two-way ANOVA	Gender	21.556	1	21.556	4.232	*P* < 0.05
	Treatment	994.629	7	142.090	27.897	*P* < 0.001
	Gender-treatment	79.974	7	11.425	2.243	*P* < 0.05
	Error Total	407.472 29070.773	80	5.093		

P<0.001 indicates significant effect at *α* = 0.001, and *P* < 0.05 indicates significant effect at *α* = 0.05.

**Table 10:** One-way and two-way analysis to test the effect of gender, treatment, and their interactions on GPx activity in rats.

Source of variation		Sum of squares	D.F.	Mean squares	*F* ratio	*P* value
One-way ANOVA	General effect					
	In between groups	381.014	15	25.401	14.032	*P* < 0.001
	Within groups	144.912	80	1.811		
	Total	525.925	95			
Two-way ANOVA	Gender	10.140	1	10.140	5.437	*P* < 0.05
	Treatment	336.466	7	48.067	25.775	*P* < 0.001
	Gender-treatment	30.130	7	4.304	2.308	*P* < 0.05
	Error Total	149.189 6250.769	80	1.865		

*P* < 0.05 indicates significant effect at *α* = 0.05.

**Table 11:** One-way and two-way analysis to test the effect of gender, treatment, and their interactions on SOD activity in rats.

Source of variation		Sum of squares	D.F.	Mean squares	*F* ratio	*P* value
One-way ANOVA	General effect					
	In between groups	212.628	15	14.175	10.941	*P* < 0.001
	Within groups	103.647	80	1.296		
	Total	316.275	95			
Two-way ANOVA	Gender	5.956	1	5.956	4.595	*P* < 0.05
	Treatment	203.057	7	29.008	22.347	*P* < 0.001
	Gender-treatment	3.406	7	0.487	0.375	*P* > 0.05
	Error Total	103.847 5047.615	80	1.298		

*P* < 0.001indicates significant effect at *α* = 0.001, and *P* < 0.05 indicates significant effect at *α* = 0.05, while *P* > 0.05 is insignificant.

**Table 12:** Protective effect of MSCs and curcumin against CFA-induced changes in LPO; GSH concentration; and GST, GPx, and SOD activities in serum of all experimental groups.

Parameter		Group							
		Normal	CMC/PBS	Curcumin	MSCs	Arthritic group	Arthritic group treated with curcumin	Arthritic group treated with MSCs	Arthritic group treated with MSCs and curcumin
LPO (nmol/mL)	Male rats	2.625 ± 0.461^a^	2.817 ± 0.399^a^	2.632 ± 0.451^a^	2.847 ± 0.313^a^	7.530 ± 1.99^b^	2.613 ± 0.351^a^	3.23 ± 2.07^a^	2.612 ± 0.397^a^
	Female rats	4.701 ± 0.440^a^	3.827 ± 0.460^a^	3.858 ± 0.504^a^	3.572 ± 0.398^a^	9.500 ± 1.002^c^	3.953 ± 0.517^d^	5.475 ± 0.404^a^	3.582 ± 0.530^a^
GSH (nmol/mL)	Male rats	8.106 ± 0.428^a^	7.468 ± 0.393^a^	8.148 ± 0.590^a^	7.858 ± 0.426^a^	3.299 ± 0.233^b^	7.425 ± 0.433^a^	7.336 ± 0.360^a^	7.753 ± 0.81^a^
	Female rats	7.375 ± 0.726^a^	7.221 ± 0.447^a^	7.566 ± 0.659^a^	7.381 ± 0.509^a^	2.088 ± 0.072^b^	6.978 ± 0.555^a^	6.900 ± 0.561^a^	7.222 ± 0.549^a^
GST (U/mL)	Male rats	18.541 ± 0.400^a^	18.496 ± 0.842^a^	18.053 ± 0.419^a^	18.453 ± 0.532^a^	5.690 ± 0.506^b^	17.755 ± 1.21^d^	16.811 ± 2.31^d^	17.970 ± 0.948^a^
	Female rats	18 ± 0.968^a^	18.034 ± 0.584^a^	18.203 ± 0.863^a^	18.880 ± 0.540^a^	11.268 ± 0.573^c^	17.853 ± 0.809^d^	18.78 ± 0.830^d^	18.39 ± 0.590^a^
GPx (U/mL)	Male rats	8.541 ± 0.400^a^	8.498 ± 0.415^a^	8.566 ± 0.761^a^	9.363 ± 0.352^a^	1.901 ± 0.227^b^	7.583 ± 0.668^d^	7.798 ± 0.589^d^	8.925 ± 0.938^a^
	Female rats	8.725 ± 0.465^a^	9.083 ± 0.550^a^	8.780 ± 0.436^a^	7.988 ± 0.837^a^	3.771 ± 0.360^c^	8.058 ± 0.281^d^	8.030 ± 0.451^d^	8.672 ± 0.436^a^
SOD (U/mL)	Male rats	7.001 ± 0.342^a^	7.446 ± 0.383^a^	7.251 ± 0.563^a^	8.156 ± 0.313^a^	3 ± 0.085^b^	7.330 ± 0.403^a^	7.253 ± 0.518^a^	6.728 ± 0.696^a^
	Female rats	7.915 ± 0.405^a^	8.058 ± 0.537^a^	7.568 ± 0.383^a^	7.855 ± 0.746^a^	3.431 ± 0.371^c^	7.638 ± 0.461^a^	7.716 ± 0.465^a^	7.925 ± 0.405^a^

Data are expressed as mean ± SE. The number of animals in each group is six. In the same column, values with the same superscript letter are similar (nonsignificant, *P* > 0.05), whereas others are significant (significant, *P* < 0.05).

## Data Availability

The raw data supporting the conclusions of this article will be made available by the corresponding author upon reasonable request.

## Abbreviations

CFA: Complete Freund's adjuvant
BM-MSCs: Bone marrow-derived mesenchymal stem cells
RA: Rheumatoid arthritis
PBS: Phosphate-buffered saline
TNF-*α*: Tumor necrosis factor-*α*
IL: Interleukin
MHC: Major histocompatibility complex
Th: T helper
ROS: Reactive oxygen species
NF-*κ*B: Nuclear factor-kappa B
CAT: Catalase
SOD: Superoxide dismutase
GPx: Glutathione peroxidase
GSH: Reduced glutathione
NOS: Nitric oxide synthase
HSCs: Hematopoietic stem cells
PDL: Programmed death ligand
GVHD: Graft-versus-host disease
FBS: Fetal bovine serum
DMEM: Dulbecco's modified Eagle's medium
g: Gram
CMC: Carboxymethyl cellulose
rpm: Rounds per minute
TBARS: Thiobarbituric acid-reactive substances
LPO: Lipid peroxidation
GST: Glutathione-S-transferase
ANOVA: Analysis of variance
H: Hour
mRNA: Messenger ribonucleic acid
CIA: Collagen-induced arthritis
IFN-*γ*: Interferon-*γ*.
